# Gastro-Cardiology: A Novel Perspective for the Gastrocardiac Syndrome

**DOI:** 10.3389/fcvm.2021.764478

**Published:** 2021-11-17

**Authors:** Robin Hofmann, Magnus Bäck

**Affiliations:** ^1^Department of Clinical Science and Education, Division of Cardiology, Karolinska Institutet, Södersjukhuset, Stockholm, Sweden; ^2^Department of Medicine Solna, Karolinska Institutet and Department of Cardiology, Karolinska University Hospital, Stockholm, Sweden

**Keywords:** gastrocardiac syndrome, *Helicobacter pylori*, cardiovascular disease, upper gastrointestinal (GI) bleeding, atherosclerosis, mortality, myocardial infarction, stroke

## Abstract

The gastrocardiac syndrome was coined originally at the beginning of the 19^th^ century to describe an alleged gastric-cardiopathy with reflux heartburn mimicking cardiac chest pain. Today, a wider perspective of gastrocardiac syndrome has emerged. First, the cardiovascular risk factor chronic systemic inflammation may reflect gastroenterological inflammatory conditions, such as inflammatory bowel disease and gastrointestinal infections, in particular, chronic *Helicobacter pylori* infection. Furthermore, since contemporary treatment of cardiovascular disease commonly includes potent antithrombotic medications, the cardiovascular benefit in terms of a decrease in the incidence of recurrent ischemic events and death needs to be carefully balanced with an increased risk of gastrointestinal bleeding. Several strategies to target chronic gastrointestinal inflammation and to diagnose and treat *Helicobacter pylori* to reduce the risk of cardiovascular events and gastrointestinal bleeding are available but residual controversy remains and large-scale gastro-cardiology trials are needed to determine the optimal treatment approaches. In perspective, the centennial gastrocardiac syndrome is more relevant than ever in a contemporary gastroenterology and cardiology setting. A collaborative subspecialty, namely Gastro-cardiology, would introduce novel unique means to study, diagnose and treat gastrocardiac conditions with the aim to reduce the risk of cardiovascular and bleeding events to improve the prognosis for gastro-cardiology patients.

## Introduction

The gastrocardiac syndrome was coined by Ludwig von Roemheld in 1913 to describe an alleged gastric-cardiopathy (1). Contemporary cardiology did however not adopt this diagnosis and reduced the gastrocardiac syndrome to define reflux heartburn mimicking cardiac chest pain. Today, modern cardiology is rapidly developing a close relation to other medical specialties. Cardio-oncology has been formalized, and for example, cardio-rheumatology and cardio-nephrology emerge as subspecialties. The frequent intersections of the roads of gastroenterologists and cardiologists in the centennium following the coining of Roemheld of gastrocardiac provide the perspective to debouch in gastro-cardiology today.

## Chronic Systemic Inflammation and Cardiovascular Disease

**Figure 1:**
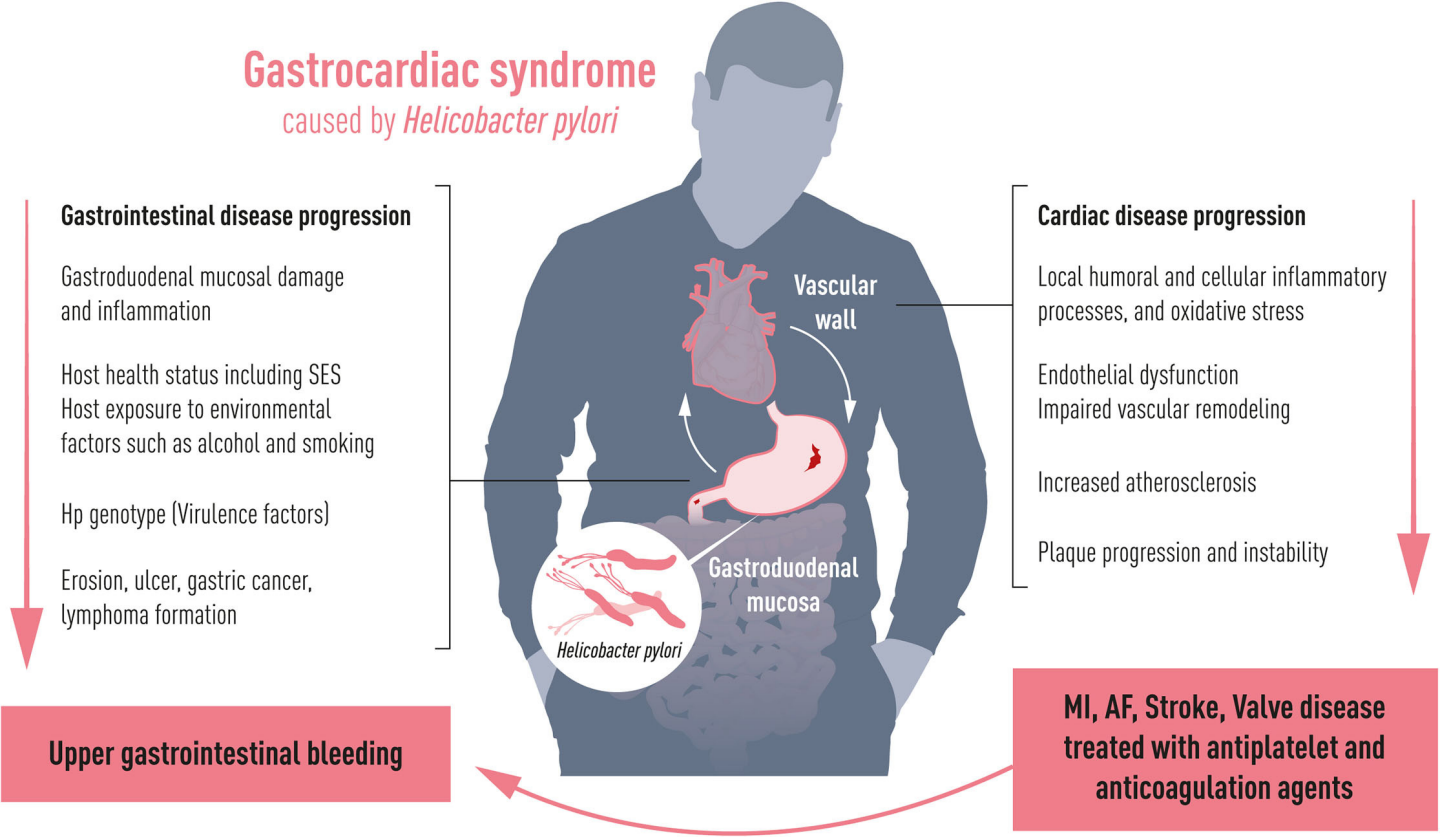
Gastrocardiac syndrome. Depending on host health including socioeconomic status (SES) and virulence factors, *Helicobacter pylori* affects the gastric mucosa causing gastroduodenal lesions with the risk to develop bleeding complications. Simultaneously, it induces chronic inflammation to the vascular wall affecting progress and degree of cardiovascular disease such as stroke, myocardial infarction (MI), atrial fibrillation (AF) and valvular heart disease. Cardiovascular diseases are commonly treated with antithrombotic drugs, which aggravate the risk of upper gastrointestinal bleeding.

Chronic systemic inflammation is a well-established cardiovascular risk factor with an underlying immune activation as a major pathophysiological driver in atherosclerosis (2). Chronic inflammation of intestinal origin from Crohn's disease and ulcerative colitis is associated with an increased risk of coronary, cerebrovascular, and peripheral artery disease (3). Trials of anti-inflammatory treatments for optimized cardiovascular prevention and controlled inflammatory bowel disease however differ substantially in terms of the putative therapeutic immune targets (4, 5). Common trial planning, follow-up, analysis, and performance are required to align the research for the optimal gastro-intestinal anti-inflammatory targets. In addition to inflammatory bowel disease, the centenarian gastrocardiac syndrome can today be extended to chronic inflammation through *Helicobacter pylori* (*H. pylori*) infection as an additional ventriculo-duodenal causal factor for atherosclerosis progression and cardiovascular events (6). An almost doubled atherothrombotic cardiovascular risk by *H. pylori* has been established over the last two decades (7). The possible mechanism linking *H. pylori* and atherosclerosis could relate to two pathways: (1) chronic inflammation caused by direct colonization of the vascular wall enhancing and disrupting atherosclerotic lesions, and (2) a systemic inflammatory response in reaction to the colonization of the gastric mucosa (6). Where the first mechanism drives the inflammatory cascade by local and humoral processes toward plaque progression and instability, the latter is influenced by the host health status (e.g., comorbidities such as diabetes) including socioeconomic factors, and host exposure to environmental factors such as smoking and alcohol, all recognized independent predictors of poor cardiovascular outcomes (Figure 1). A common gastro-cardiology fight to dampen inflammation can be further encouraged by *H. pylori* being one of the most common chronic infections in the world with an estimated global prevalence of 50% (ranging from 10% in Northern Europe to 80% in Africa) (8) and the potential that lowering inflammation reduces the burden of the most common mortality cause from cardiovascular disease.

## Gastrointestinal Bleeding and Cardiovascular Disease

Gastrointestinal bleedings have been a common problem in cardiology starting from the discovery of Heyde's syndrome in the past century. Advances since then have deciphered the mechanisms being that blood passage through a stenotic aortic valve increases shear stress to deplete von Willebrand factor. The acquired von Willebrand factor deficiency represents a reverse causality with the heart causing bleedings from gastrointestinal angiodysplasias in patients with calcific aortic valve stenosis (9). During the last decades, the prognosis for patients with cardiovascular disease, including ischemic heart disease, valvular heart disease, atrial fibrillation, or stroke has remarkably improved. However, the implementation of evidence-based therapies, in particular, the use of antithrombotic treatment presents gastro-cardiological consequences (10). Indeed, the cardiovascular benefit in terms of a decrease in the incidence of recurrent ischemic events and death is counterbalanced by an increase in hemorrhagic complications (11), in particular, from the gastrointestinal tract (12, 13). These typically present as upper gastrointestinal bleedings (UGIB), ranging from a 2-fold increase with low dose aspirin, up to 7-fold with dual anti-platelet treatment, and by a factor of 10 if anticoagulants are co-administered (10). The UGIB complications are not only the direct source for increased morbidity, mortality (14), and medical care costs but may also lead to increased risk of recurrent cardiovascular events due to discontinuation of antithrombotic drugs. Chronic active infection with *H. pylori* may be a common gastric orchestrator of chronic inflammation (6) and UGIB complications in cardiology (10).

Numerous studies over the last two decades have investigated a possible link between *H. pylori* infection and atherothrombotic cardiovascular syndromes and found an association with a two-fold increased risk (7, 15).

While previous studies of *H. pylori* eradication in cardiology aimed to reduce cardiovascular events, the effects of *H. pylori* eradication on reducing cardiological bleeding complications have been somewhat overlooked (16). Several strategies to reduce the risk of UGIB are available. First, the cardiology perspective is a personalized antithrombotic therapy through shortened duration (17) and/or de-escalation (18) based on clinical features and/or risk scores (19–22). Second, the gastroenterologist perspective complies chronic inhibition of gastric acid secretion by proton pump inhibitors (PPI), which are currently recommended in high-risk individuals (defined as a history of gastric ulcer/bleeding, anticoagulant therapy, chronic non-steroidal anti-inflammatory drugs/corticoid steroid use, or two or more of age ≥65 years, dyspepsia, gastro-esophageal reflux disease, *H. pylori* infection or chronic alcohol use) (23) to decrease bleeding risk during DAPT post-AMI (24). However, the net benefit of long-term PPI treatment is unresolved. Adverse events include higher rates of chest infections, dementia, cardiovascular events, and chronic kidney disease (24). Further, *H. pylori* eradication which may achieve similar benefits in infected patients while avoiding the side effects and medication costs associated with long-term PPI use (25), is recommended in guidelines, notably, by expert opinion (8, 26). Concerning the mode of *H. pylori* detection, both invasive (gastroscopy-based) and non-invasive (serology, urea breath test [UBT], feces antigen) methods are established (8). As invasive tests imply obvious drawbacks in patients with CV disease with concomitant antithrombotic therapy, the majority of previous studies were based on serological testing to detect *H. pylori*. However, serology does not allow distinction between active and prior *H. pylori* infection, which encompasses diagnostic difficulties, especially from a clinical perspective regarding eradication therapy. Non-invasive screening for active *H. pylori* can be performed with high accuracy (sensitivity 96% and specificity 93%) by UBT (27), currently the recommended diagnostic tool (8). No contemporary data using this mode of detection were available until recently when it was shown in a Swedish multicenter prospective cohort study of 310 consecutive AMI patients that active *H. pylori* diagnosed by UBT were prevalent in 20% of the patients (16), in agreement with previous findings twice as common as in the overall Swedish population with an *H. pylori* prevalence estimated at 11% (28). Importantly, *H. pylori* screening and treatment were feasible in the clinical routine during MI hospitalization. Currently, it is still under debate whether eradication therapy alone is sufficient to prevent recurrent UGIB or if long-term PPI treatment nonetheless remains necessary (29). Thus, gastro-cardiology is needed to address the net benefit of long-term treatments, adverse events, and medication costs of antithrombotic and antiacid treatments, and, critically, to determine how to handle *H. pylori* diagnosis and eradication.

## Current Frontier of Clinical Gastro-Cardiology Research

To date, the residual controversy on how to handle the risk of UGIB in patients with cardiovascular disease urges the need for randomized gastro-cardiology trials. Recently, the Helicobacter eradication aspirin trial (HEAT), a double-blind, placebo-controlled, randomized trial of the effects of *H. pylori* eradication on subsequent ulcer bleeding in infected individuals taking aspirin daily was completed enrolling 30,000 patients (30). The HELicobacter Pylori screening to prevent gastrointestinal bleeding in patients with acute myocardial infarction trial based on the Swedish Web System for Enhancement and Development of Evidence-based Care in Heart Disease Evaluated According to Recommended Therapies (HELP-SWEDEHEART, ClinicalTrials.gov Identifier: NCT05024864) is a cluster-randomized, registry-based clinical trial using SWEDEHEART (31) and other national registries as a trial platform for patient enrollment and data collection. The primary objective is to determine whether systematic screening for *H. pylori* in patients after AMI and subsequent eradication therapy significantly reduces the risk of rehospitalization for UGIB whereas secondary objectives evaluate the incidence of cardiovascular endpoints (rehospitalization for AMI, heart failure, atrial fibrillation, and stroke; cardiovascular and all-cause mortality). Patient enrollment is expected to start in November 2021.

## Conclusion

In perspective, the centennial gastrocardiac syndrome is more relevant than ever in a contemporary gastroenterology and cardiology setting. A collaborative subspecialty to improve diagnosis and treatment of gastrocardiac conditions and to reduce the cardiovascular risk and complications of ischemic-, arrhythmic- and valvular heart diseases would introduce novel unique means to improve the outcomes of gastro-cardiology patients.

## Data Availability Statement

The original contributions presented in the study are included in the article/supplementary material, further inquiries can be directed to the corresponding author/s.

## Author Contributions

RH and MB: concept and design, critical revision of the manuscript for important intellectual content, and drafting of the manuscript. All authors contributed to the article and approved the submitted version.

## Funding

RH was supported by the Swedish Heart-Lung Foundation [grant number, HLF 2018-0187]; the Swedish Research Council [grant number, 2019-00414]; and the Region Stockholm (clinical postdoctoral appointment) [grant number, K 2017-4577]. The funding organizations had no role in the preparation, review, or approval of the manuscript; and decision to submit the manuscript for publication.

## Conflict of Interest

The authors declare that the research was conducted in the absence of any commercial or financial relationships that could be construed as a potential conflict of interest.

## Publisher's Note

All claims expressed in this article are solely those of the authors and do not necessarily represent those of their affiliated organizations, or those of the publisher, the editors and the reviewers. Any product that may be evaluated in this article, or claim that may be made by its manufacturer, is not guaranteed or endorsed by the publisher.

## References

[1] Roemheld L. Der gastro-kardiale Symptomenkomplex, eine besondere Form sog. Herzneurose Fortschr Med. (1913) 3:57.

[2] Back M Yurdagul A Tabas I Oorni K Kovanen PT. Inflammation and its resolution in atherosclerosis: mediators and therapeutic opportunities. Nat Rev Cardiol. (2019) 16:389–406. 10.1038/s41569-019-0169-2 30846875 PMC6727648

[3] Wu H Hu T Hao H Hill M Xu C Liu Z. Inflammatory bowel disease and cardiovascular diseases: a concise review. Eur Heart J Open. (2021). 10.1093/ehjopen/oeab029 PMC9242064 35919661

[4] Sands BE Colombel JF Ha C Farnier M Armuzzi A Quirk D. Lipid profiles in patients with ulcerative colitis receiving tofacitinib-implications for cardiovascular risk and patient management. Inflamm Bowel Dis. (2021) 27:797–808. 10.1093/ibd/izaa227 32870265 PMC8128390

[5] Ridker PM Everett BM Thuren T MacFadyen JG Chang WH Ballantyne C. Antiinflammatory therapy with canakinumab for atherosclerotic disease. N Engl J Med. (2017) 377:1119–31. 10.1056/NEJMoa1707914 28845751

[6] Budzynski J Kozinski M Klopocka M Kubica JM Kubica J. Clinical significance of Helicobacter pylori infection in patients with acute coronary syndromes: an overview of current evidence. Clin Res Cardiol. (2014) 103:855–86. 10.1007/s00392-014-0720-4 24817551

[7] Fang Y. Fan C. Xie H. Effect of Helicobacter pylori infection on the risk of acute coronary syndrome: a systematic review and meta-analysis. Medicine (Baltimore). (2019) 98:e18348. 10.1097/MD.0000000000018348 31852134 PMC6922357

[8] Malfertheiner P Megraud F O'Morain CA Gisbert JP Kuipers EJ Axon AT. Management of Helicobacter pylori infection-the Maastricht V/Florence Consensus Report. Gut. (2017) 66:6–30. 10.1136/gutjnl-2016-312288 27707777

[9] Pawelzik SC. Back M. Von willebrand factor's vascular crossroad. Cardiovasc Res. (2021). [Epub ahead of print]. 10.1093/cvr/cvab253 34375395

[10] Hellstrom PM Benno P Malfertheiner P. Gastrointestinal bleeding in patients with Helicobacter pylori and dual platelet inhibition after myocardial infarction. Lancet Gastroenterol Hepatol. (2021) 6:684–5. 10.1016/S2468-1253(21)00192-8 34391514

[11] Valgimigli M Costa F Lokhnygina Y Clare RM Wallentin L Moliterno DJ. Trade-off of myocardial infarction vs. bleeding types on mortality after acute coronary syndrome: lessons from the Thrombin Receptor Antagonist for Clinical Event Reduction in Acute Coronary Syndrome (TRACER) randomized trial. Eur Heart J. (2017) 38:804–10. 10.1093/eurheartj/ehw525 28363222 PMC5837470

[12] Valgimigli M Costa F Lokhnygina Y Clare RM Wallentin L Moliterno DJ. Characterising and predicting bleeding in high-risk patients with an acute coronary syndrome. Heart. (2015) 101:1475–84. 10.1136/heartjnl-2014-307346 26109589

[13] Magnani G Ardissino D Im K Budaj A Storey RF Steg PG. Predictors, type, and impact of bleeding on the net clinical benefit of long-term ticagrelor in stable patients with prior myocardial infarction. J Am Heart Assoc. (2021) 10:e017008. 10.1161/JAHA.120.017008 33559485 PMC7955333

[14] Sarajlic P Simonsson M Jernberg T Back M Hofmann R. Incidence, associated outcomes, and predictors of upper gastrointestinal bleeding following acute myocardial infarction: a SWEDEHEART-based nationwide cohort study. Eur Heart J Cardiovasc Pharmacother. (2021). [Epub ahead of print]. 10.1093/ehjcvp/pvab059 34423350 PMC9366628

[15] Shindler-Itskovitch T Chodick G Shalev V Muhsen K. Helicobacter pylori infection and prevalence of stroke. Helicobacter. (2019) 24:e12553. 10.1111/hel.12553 30431685

[16] Warme J Sundqvist M Mars K Aladellie L Pawelzik SC Erlinge D. Helicobacter pylori screening in clinical routine during hospitalization for acute myocardial infarction. Am Heart J. (2021) 231:105–9. 10.1016/j.ahj.2020.10.072 33144087

[17] Wilson SJ Newby DE Dawson D Irving J Berry C. Duration of dual antiplatelet therapy in acute coronary syndrome. Heart. (2017) 103:573–80. 10.1136/heartjnl-2016-309871 28249994 PMC5529971

[18] Mehran R Baber U Sharma SK Cohen DJ Angiolillo DJ Briguori C. Ticagrelor with or without Aspirin in High-Risk Patients after PCI. N Engl J Med. (2019) 381:2032–42. 10.1056/NEJMoa1908419 31556978

[19] Subherwal S Bach RG Chen AY Gage BF Rao SV Newby LK. Baseline risk of major bleeding in non-ST-segment-elevation myocardial infarction: the CRUSADE (Can Rapid risk stratification of Unstable angina patients Suppress ADverse outcomes with Early implementation of the ACC/AHA Guidelines) Bleeding Score. Circulation. (2009) 119:1873–82. 10.1161/CIRCULATIONAHA.108.828541 19332461 PMC3767035

[20] Yeh RW Secemsky EA Kereiakes DJ Normand SL Gershlick AH Cohen DJ. Development and validation of a prediction rule for benefit and harm of dual antiplatelet therapy beyond 1 year after percutaneous coronary intervention. J Am Med Assoc. (2016) 315:1735–49. 10.1001/jama.2016.3775 27022822 PMC5408574

[21] Costa F van Klaveren D James S Heg D Raber L Feres F. Derivation and validation of the predicting bleeding complications in patients undergoing stent implantation and subsequent dual antiplatelet therapy (PRECISE-DAPT) score: a pooled analysis of individual-patient datasets from clinical trials. Lancet. (2017) 389:1025–34. 10.1016/S0140-6736(17)30397-5 28290994

[22] Urban P Mehran R Colleran R Angiolillo DJ Byrne RA Capodanno D. Defining high bleeding risk in patients undergoing percutaneous coronary intervention: a consensus document from the Academic Research Consortium for High Bleeding Risk. Eur Heart J. (2019) 40:2632–53. 10.1093/eurheartj/ehz372 31116395 PMC6736433

[23] Collet JP Thiele H Barbato E Barthelemy O Bauersachs J Bhatt DL. 2020 ESC Guidelines for the management of acute coronary syndromes in patients presenting without persistent ST-segment elevation. Eur Heart J. (2021) 42:1289–367. 10.1093/eurheartj/ehaa909 32860058

[24] Sehested TSG Carlson N Hansen PW Gerds TA Charlot MG Torp-Pedersen C. Reduced risk of gastrointestinal bleeding associated with proton pump inhibitor therapy in patients treated with dual antiplatelet therapy after myocardial infarction. Eur Heart J. (2019) 40:1963–70. 10.1093/eurheartj/ehz104 30851041

[25] Gisbert JP Khorrami S Carballo F Calvet X Gene E Dominguez-Munoz E. Meta-analysis: helicobacter pylori eradication therapy vs. antisecretory non-eradication therapy for the prevention of recurrent bleeding from peptic ulcer. Aliment Pharmacol Ther. (2004) 19:617–29. 10.1111/j.1365-2036.2004.01898.x 15023164

[26] Chey WD Leontiadis GI Howden CW Moss S. FACG clinical guideline: treatment of helicobacter pylori infection. Am J Gastroenterol. (2017) 112:212–39. 10.1038/ajg.2016.563 28071659

[27] Gisbert JP Khorrami S Carballo F Calvet X Gene E Dominguez-Munoz E. Accuracy of urea breath test in Helicobacter pylori infection: meta-analysis. World J Gastroenterol. (2015) 21:1305–14. 10.3748/wjg.v21.i4.1305 25632206 PMC4306177

[28] Agreus L Hellstrom PM Talley NJ Wallner B Forsberg A Vieth M. Towards a healthy stomach? Helicobacter pylori prevalence has dramatically decreased over 23 years in adults in a Swedish community United European. Gastroenterol J. (2016) 4:686–96. 10.1177/2050640615623369 27733911 PMC5042307

[29] Vergara M Catalan M Gisbert JP Calvet X. Meta-analysis: role of Helicobacter pylori eradication in the prevention of peptic ulcer in NSAID users. Aliment Pharmacol Ther. (2005) 21:14114–8. 10.1111/j.1365-2036.2005.02444.x 15948807

[30] Dumbleton JS Avery AJ Coupland C Hobbs FD Kendrick D Moore MV. The helicobacter eradication aspirin trial (HEAT): a large simple randomised controlled trial using novel methodology in primary care. EBioMedicine. (2015) 2:1200–4. 10.1016/j.ebiom.2015.07.012 26501118 PMC4588401

[31] Jernberg T Attebring MF Hambraeus K Ivert T James S Jeppsson A. The Swedish Web-system for enhancement and development of evidence-based care in heart disease evaluated according to recommended therapies (SWEDEHEART). Heart. (2010) 96:1617–21. 10.1136/hrt.2010.198804 20801780

